# Differences in Metabolic Control Between Different Insulin Use Patterns in Pediatric Patients with Type 1 Diabetes Through Intermittent Glucose Monitoring

**DOI:** 10.3390/diseases13080254

**Published:** 2025-08-09

**Authors:** Rocio Porcel-Chacón, Leopoldo Tapia-Ceballos, Ana-Belen Ariza-Jimenez, Ana Gómez-Perea, José Manuel Jiménez-Hinojosa, Juan-Pedro López-Siguero, Isabel Leiva-Gea

**Affiliations:** 1 Universitary Costa del Sol Hospital A-7, Km 187, 29603 Málaga, Spain; rocio.porcel.86@gmail.com; 2 Regional University Hospital of Malaga, Avda Arroyo de los Angeles, s/n, 29009 Málaga, Spain; leotapiaceb@hotmail.com (L.T.-C.); gomezpereana@gmail.com (A.G.-P.); jmjhinojosa@hotmail.com (J.M.J.-H.); isabeleiva@hotmail.com (I.L.-G.); 3 Institute of Biomedicine of Malaga (IBIMA), C. Severo Ochoa, 35, Campanillas, 29590 Málaga, Spain; 4 Pediatric Endocrinology Service, Reina Sofia University Hospital, Avda. Menendez Pidal, s/n, 14004 Cordoba, Spain; 5 Growth Study Group, Pediatric Endocrinology and Nutrition, Maimonides Institute of Biomedical Research of Cordoba, Av. Menendez Pidal, s/n, 14004 Cordoba, Spain; 6 Department of Pediatrics, University of Cordoba, Avda Menendez Pidal, 7, 14004 Cordoba, Spain; 7 Department of Biomedicine and Dentistry, Faculty of Biomedical Sciences and Sports, Universidad Europea de Andalucía, 29010 Málaga, Spain

**Keywords:** Type 1 Diabetes Mellitus, continuous subcutaneous insulin infusion, multiple doses of insulin, pediatric population, intermittent continuous glucose monitoring

## Abstract

Introduction: In healthcare centers with limited resources, or for patients who prefer to make continuous changes in their treatment themselves and do not want to rely solely on technology, intermittent glucose monitoring (isCGM) with an insulin pump is a viable option that warrants further study. Material and methods: prospective single-center study that collected data at 3 months and after isCGM implantation in pediatric patients with Type 1 diabetes, categorized according to their insulin regimen. Results: We found statistically significant differences in the time in range (TIR) between 70 and 180 mg/dl at 3 months after using the sensor (*p* = 0.017), although these differences were not maintained at 1 year (*p* = 0.064). When stricter TIRs (70–140 mg/dl) were analyzed, statistically significant differences were observed at 3 months (*p* = 0.01) and at 1 year (*p* = 0.018) in favor of patients using CSII. While 75% of the patients in the CSII group had good control with HbA1c < 7% after one year of sensor use, only 34.6% in the MDI group achieved these values. However, the CSII group presented a higher coefficient of variation (62.31% at 3 months and 43.08% at 1 year) (*p* = 0.02), and a higher number of hypoglycemic episodes (7.38% and 7.32%, respectively) (*p* = 0.016). The CSII group also had a higher number of capillary blood glucose measurements at the beginning of the study (8.32/day) (*p* = 0.249), but this number became similar between both groups after one year. Conclusions: We found statistically significant differences in favor of CSII over MDI in terms of metabolic control after one year of isCGM use. However, the TIR values were still below the range considered to be indicative of good control. These findings lead us to question whether CSII should be initially considered in specific cases where HCL is not possible, or if it would be more effective to wait until the patient is ready, or the necessary resources are available to start directly CSII integrated in a closed loop system.

## 1. Introduction

Type 1 diabetes mellitus (DM1) is one of the most common chronic diseases in childhood. Its onset at an early age makes the appearance of short- and long-term frequent complications [1].

The Diabetes Control and Complications Trial (DCCT) showed that those patients who maintained HbA1c levels closer to those without diabetes had a lower incidence of microvascular and macrocardiovascular complications. Additionally, the study revealed that the initial metabolic control influenced the long-term clinical evolution, which is known as “metabolic memory” [2].

Therefore, achieving a glycosylated hemoglobin (HbA1c) level closer to that of individuals without diabetes is crucial. This can be achieved through optimizing treatments, including intensive insulin therapy, in the form of multiple doses of insulin (MDI) and continuous infusion of subcutaneous insulin (CSII), both of which have been proven effective in childhood [3,4].

Studies comparing MDI and CSII in children and adults have shown a decrease in HbA1c levels in patients using CSII, with a reduced need for insulin. The percentage of hypoglycemia was similar in both groups, with no statistically significant differences [5,6,7,8].

The objectives of good disease control based on HbA1c levels vary depending on the guidelines consulted [1,9] (Table 1). However, all guidelines agree on the need to individualize treatment goals for each patient.

The continuous development of technology has led to the emergence of different continuous glucose monitoring (CGM) systems, which measure blood glucose at the level of interstitial fluid. There are two monitoring modalities, intermittent glucose monitoring (isCGM), where the patient must bring the blood glucose reader close to the sensor, in order not to miss information, and real-time continuous monitoring (rtCGM), in which the information is saved automatically [10].

In recent years, there has been an increase in the use of isCGM, providing more information on glycemic variability throughout the day and on different days, as well as acute events of hypo- and hyperglycemia. This has resulted in a reduction in the number of capillary blood glucose measurements, as these systems do not always require calibration and can provide blood glucose levels without the use of blood glucose strips. The use of isCGM has been shown to be effective in achieving a significant reduction in HbA1C values in both children and adults [11].

Several attempts have been made to agree on the interpretation of the different parameters obtained from the reading of continuous glucose monitoring systems, as discussed at the ATTD (Advanced Technologies and Treatments for Diabetes) congress [10] (Table 2).

The rapid development of continuous glucose sensor technology has allowed for swift progress toward a hybrid closed-loop system (HCL). This is an insulin delivery system that automatically adjusts basal and bolus insulin delivery based on sensor glucose to maintain blood glucose levels as close to a specific target as possible. This possibility does not currently exist with isCGM [12].

In 2020, The CORRIDA Randomized Controlled Trial was published, in which the efficacy of obtaining more optimal metabolic control in adult patients with DM1 treated with CSII was studied, comparing isCGM (FreeStyle Libre) with rtCGM (Guardian Connect Mobile). The results showed the superiority of rtCGM in reducing hypoglycemia (8.3% vs. 5.4%, *p* = 0.0062) and improving time in range (TIR) (61.4% vs. 65.4%, *p* = 0.28) [13].

Another study conducted in 2019 in patients between 5 and 16 years of age undergoing CSII treatment analyzed the difference between isCGM (FreeStyle Libre) and rtCGM (Monitor Elite with Minimed 640G, SmartGuard Technology). They compared isCGM parameters in the last month to rtCGM parameters at 3 and 6 months, finding a lower coefficient of variation (CV) (46.2% vs. 38.4% and 36.4%, *p* = 0.000) and a lower percentage of hypoglycemia under 70 mg/dl (7.4% vs. 1.6% and 1.5%, *p* = 0.000) in patients who used rtCGM [14]. In line with this, in 2022, Babiker et al. conducted a retrospective comparison of CSII with MDI in achieving glycemic control in youths with Type 1 diabetes mellitus (T1DM) using isCGM, and the results in the CSII cohort were likewise superior [15].

Similarly, in 2022, Serné et al. conducted a study in adults with the aim of comparing the long-term cost-effectiveness of the MiniMed 670G HCL system versus isCGM plus MDI or CSII in the Netherlands. They concluded that the MiniMed 670G system is cost-effective compared to isCGM plus MDI or CSII [16].

In 2020, Breton et al. conducted a comparative study in children using CSII. One group used an HCL system (T:slim X2 insulin pump with Control-IQ Technology and rtCGM, Dexcom G6) and another used CSII with isCGM (FreeStyle Libre). After 16 weeks, the closed-loop group showed a higher time in range (TIR) of 67% compared to 55% in the control group (*p* < 0.001). Although the HCL group showed a decrease in HbA1c of 0.4%, it was not statistically significant [17].

However, the HCL system may not be available for all patients with diabetes or some patients may not want to use it. In healthcare centers with limited resources or for patients who prefer to make continuous changes in their treatment themselves, isCGM with an insulin pump may be a viable option.

While clinical trials using rtCGM have established associations between the risk of hypoglycemic and hyperglycemic episodes and various glycometric indicators, real-world assessments, particularly with isCGM, are underexplored [18]. Therefore, it is necessary to further investigate the measurements of this modality.

All of this led us consider what differences could be assumed in the pediatric population based on intermittent glucose sensor measurements and the type of therapy used: CSII vs. MDI. This is especially relevant in the context of economic and resource limitations of the healthcare system, which do not allow us to provide HCL to all users with diabetes in our area.

## 2. Material and Methods

An ambispective study was conducted before and after the implantation of intermittent scanning continuous glucose monitoring (isCGM) in a tertiary care hospital. So, both retrospective and prospective approaches were combined in a cohort study.

### 2.1. Study Design and Participants

The study was carried out in accordance with the regulations of the Official Bulletin of the Andalusian Government (BOJA), in the organization of the Andalusian Health Service (SAS) for the inclusion of continuous glucose monitoring in the services of the Andalusian Public Health and financed by the project of the Ministry of Health of Andalusia (PIGE 0533-2019).

The inclusion criteria were as follows: presence of DM1 with a disease duration of more than one year, age between 4 and 18 years at the beginning of the study, and no previous experience in the use of isCGM.

Exclusion criteria were previous use of isCGM or any other monitoring of interstitial blood glucose, and disease duration of less than one year.

The follow-up period was 1 year with evaluations at 3 months and at the end of the study at one year.

Patients were divided into five groups based on their HbA1c levels after one year of using isCGM and the treatment used.

Throughout the study, adherence to insulin therapy, diet, and isCGM scanning frequency was evaluated and emphasized in medical and nursing consultations every 3 months. All of these factors were kept within objectives during the year of follow-up to avoid confounding factors in the results.

Data were extracted using the isCGM platform, at three months and one year after implantation of isCGM in the arm, taking into account the last 14 days of its use before attending the consultation. The parameters collected were those accepted in the consensus guidelines on the interpretation of CGM: TIR, average number of scans per day, CV, which assess how much glucose levels fluctuate around the average, regardless of the absolute glucose level, and time below range (TBR) [4,5,9]. In addition to these, gender, age, and the number of capillary blood glucose measurements performed before sensor implantation and after one year of use were analyzed.

TIR is defined as the time in which the blood glucose levels are between 70 and 180 mg/dl, with some occasions using a stricter measure of TIR between 70 and 140 mg/dl.

Patient data were analyzed according to whether they used insulin in the form of a continuous subcutaneous insulin infusion system (CSII) or multiple-dose insulin (MDI).

Likewise, capillary HbA1c was also obtained one year after using isCGM, determined through a capillary blood sample using the DCA Vantage analyzer system (immunoassay technique), carried out in the Diabetes Consultation.

Quality of life (QoL) was evaluated at 3 months and 1 year of follow-up using the PedsQL 3.2 Diabetes Module for Children, Adolescents, and Young Adults.

### 2.2. Statistic Analysis

An analysis of the results was performed using different tests depending on the nature of the data. In the case of two data samples, if the data were normal, Student’s t test was used, if they were not normal, the Wilcoxon rank test was used. Normality and homoscedasticity have been verified using the Anderson–Darling (DA) and Fligner–Killen (FK) tests, respectively.

The IBM SPSS Statistics version 22 statistical package (annual educational license from the University of Malaga) was used for the analysis.

## 3. Results

One hundred ninety-one pediatric patients with DM1 under treatment with intensive insulin therapy were recruited. One hundred fifty-five of them with multiple doses of insulin and 36 patients with continuous subcutaneous insulin infusion systems.

1. Sex.

Almost half of the patients in each treatment group were male (*p* = 0.740). (Table 3).

2. Age.

The mean age in the MDI group was 11.2 years, while in the CSII group it was younger (10.78 years) (*p* = 0.323) (Table 3).

3. Time in range.

In our study, we found a significantly higher percentage of time in range with CSII versus MDI in the IR (both 70–180 mg/dl and 70–140 mg/dl) 3 months after the use of the sensor (*p* = 0.017), and in the TIR 70–140 mg/dl at one year of sensor use (*p* = 0.010) (Table 4).

4. Number of scans

The number of sensor scans that the patient performs did not show statistically significant differences either at 3 months or at one year in patients with CSII versus MDI (*p* = 0.3) (Table 4).

5. Coefficient of variation

Statistically significant differences were demonstrated at 3 months in the CV values between both groups, being higher in the CSII group (62.31% versus 40.58%) (*p* = 0.02). These differences were less at one year of follow-up (CSII group 43.08% vs. MDI group 40.36%) but remained statistically significant (*p* = 0.02) (Table 4).

6. Time under range

The percentage of time in hypoglycemia was higher in the group treated with CSII at 3 months (*p* = 0.016) and at one year of follow-up (*p* = 0.03), obtaining statistically significant differences in both cut-off points, 55 and 70 mg/dl (Table 4).

7. Categorization according to the level of HbA1c after one year of sensor use

When categorizing according to HbA1c, statistically significant differences were found after one year of use (*p* = 0.013).

While 75% of the patients in the CSII group had good control with HbA1c < 7% at one year of sensor use, only 34.6% in the MDI group were at those values. (Table 5).

During the evolution inside each group, MDI experienced an increment in the group with hbA1c between 7 and 7.5% at 1 year with respect to basal controls (*p* = 0,01), and diminished groups before 7%, although without statistical significance (*p* = 0.99), while the CSII group incremented the patients in the 6.5–7% range, although without statistical significance (*p* = 0.11).

8. Number of capillary blood glucose measurements before and after the use of isCGM

In our study, we observed statistically significant differences in the number of capillary blood glucose measurements performed in the two study groups before the introduction of the interstitial glucose sensor (*p* < 0.01). A higher number of blood glucose tests were performed in the CSII group (8.32/day) than in the MDI group (6.77/day).

One year after using the sensor, no differences were observed between the two groups, with both groups experiencing a marked decrease in the number of capillary blood glucose measurements performed (*p* = 0.249). (Table 6).

9. Qol.

We observed, according to the results of quality of life surveys specific to diabetes (PedsQL 3.2) in both groups, higher scores at 3 months and one year of isCGM use, although higher in CSII group, with a statistically significant difference.

## 4. Discussion

In our study, initially, there were no differences between the two groups (CSII vs. MDI) when categorizing patients according to their HbA1c values. However, after one year of using a sensor, we found significant differences (*p* = 0.013), with a higher percentage of patients with good control in the CSII group based on HbA1c levels (NICE < 6.5%, ISPAD < 7%) [1,9].

At the beginning, we also noticed a statistically significant difference in the number of capillary blood glucose tests between the two groups, with the CSII group having a higher number of tests. However, this difference was not maintained after one year of using the sensor, indicating that the improvement in HbA1c or TIR achieved cannot be solely attributed to the number of capillary blood glucose tests performed [19]. This could be due to the fact that patients using a pump tend to have a more involved profile with their diabetes and usually receive extra training on how to correctly use the insulin delivery device.

When looking at the number of scans, we did not find any differences between the groups at 3 months or one year.

However, we found statistically significant differences in TIR between 70 and 180 mg/dl at 3 months (*p* = 0.017), but these differences were not maintained at one year (*p* = 0.064). These differences suggest that patients using CSII may have a more optimal metabolic control based on a higher TIR. However, it is important to note that the TIR values in patients with CSII, both at 3 months (47%, SD ± 15) and one year (54%, SD ± 12.7), are still far from the recommended TIR > 70% for good metabolic control according to the consensus CGM [4,9].

When we analyzed stricter TIR values of 70–140 mg/dl, we found statistically significant differences at 3 months (*p* = 0.01) and one year (*p* = 0.018) in favor of patients using CSII. These results are consistent with previous studies [12,18] that have also reported similar TIR values for patients using CSII and isCGM (47.1–29.2% in the literature vs. 54.78% with TIR 70–180 mg/dl and 35.63% with TIR 70–140 mg/dl, in our patients) [15,18].

Having said that, it is important to note that both groups failed to achieve ideal glycemic targets. This raises the question of whether there is limited clinical benefit from either regimen with isCGM alone in this cohort. However, there are several studies that support the fact that most children and adolescents with diabetes have suboptimal glycemic control regardless of the type of sensor and insulin delivery device. Cherubini et al. found that only 8.3% of participants using MDI-isCGM and 28.1% using CSII-rtCGM met the TIR > 70% target [20]. This could be due to the demanding nature of the disease, the presence of long-term complications, self-care capacity, and associated costs [20].

Our findings also showed that patients using CSII had better results in terms of quality of life (QoL), although it is worth noting that the literature suggests that diabetes-related distress improves after starting isCGM in both modalities of insulin administration [21].

In terms of TBR (glycemia less than 70 mg/dl), we also found significant differences between the groups at 3 months and one year, with the CSII group having a higher percentage of TBR. However, only the MDI group met the objective of good metabolic control (TBR < 5%) at one year of follow-up.

It is important to mention that although isCGM generally does not offer real-time alerts like real-time rtCGM or HCL systems, it is possible to set a hypoglycemia alarm that notifies the user when their glucose levels fall below a predefined threshold. This could avoid differences that potentially influence hypoglycemia outcomes across groups.

Our data regarding CV and hypoglycemia in patients with CSII after one year of using the flash system are similar to those found in the literature [13,17,22]. This is consistent with other studies that have shown significant improvements in HbA1c and hypoglycaemia unawareness in adults with T1D using isCGM [21]. These findings can be extrapolated to our sample.

Pediatric patients with DM1 who used isCGM in conjunction with CSII showed a CV of 46.2% (compared to our patients’ 43%) and a TBR of 7.4% (compared to our patients’ 7.32%). It is striking that the CV remained relatively unchanged in the MDI group at 3 months and one year of sensor use, while the CSII group saw a decrease in CV from 62.31% to 43.08%. However, it is important to note that none of the groups achieved a CV below 36%, which is considered as a marker of good control.

As a limitation, it should be mentioned that there was no control group to compare the isCGM parameters before implantation, and there were no baseline blood glucose monitoring data.

Additionally, factors such as socioeconomic status, education level, family support, and diabetes duration were not collected or adjusted for, despite their potential to bias pediatric diabetes outcomes.

Furthermore, it is possible that patients were not randomly assigned to the CSII or MDI groups, which raises concerns about selection bias, as patients with better diabetes management skills may have been preferentially placed on CSII.

Finally, the single-center nature and small sample size of the CSII group could have been additional limitations in the generalizability of the findings to diverse healthcare settings and populations, and may limit the generalizability of the findings to across diverse healthcare settings and populations, as well as the statistical power of the study.

However, the strength of our study is that that we had a larger sample size and longer follow-up period compared to most other published studies.

In conclusion, our findings after one year of using isCGM are consistent with those reported in the literature, but they are insufficient in achieving the goal of good metabolic control.

These observations, supported by the existing literature, lead us to question whether CSII should be initially implemented with isCGM in specific cases where HCL is not possible, or if it would be more effective to wait until the patient is ready or the necessary resources are available to start CSII integrated into a closed-loop system, although this may involve a period of waiting while the patient remains on MDI.

## Figures and Tables

**Table 1:** Reference values for blood glucose and HbA1c according to different societies.

	NICE	ISPAD	ADA
Preprandial blood glucose (mg/dl)	70–126	70–130	90–130
Postprandial blood glucose (mg/dl)	90–162	90–180	
Glycemia before-bedtime (mg/dl)	70–126	80–140	90–150
HbA1C (%)	≤6.5	<7	<7.5

mg: milligrams; dl: deciliter; HbA1c glycosylated hemoglobin; NICE National Institute for Health and Care Excellence; ISPAD International Society for Pediatric and Adolescent Diabetes; ADA American Diabetes Association.

**Table 2:** Standardized CGM parameters in the ATTD 2019 consensus and their target values for Type 1 diabetes.

Variable	Aims
1. Number of days of isCGM	14 days
2. Percentage of active isCGM time	>70%
3. Glucose Mean/Standard Deviation	<154 mg/dl/<29%
4. Glucose Management Indicator (GMI)/estimated HbA1C	<7%
5. Glucose variability (CV variation coefficient) (%)	<36%
6. Time in range or in range rate from 70 to 180 mg/dl (% of time)	>70%/>16 h 48 min
7. Time over range >180 mg/dl (% of time). Level 1 hyperglycemia	<25%/<6 h
8. Time over range >250 mg/dl (% of time). Level 2 hyperglycemia	<5%/<1 h 12 min
9. Time below range <70 mg/dl (% of time). Level 1 hypoglycemia	<4%/<1 h
10. Time below range <54 mg/dl (% of time). Level 2 hypoglycemia	<1%/<15 min

CGM Continuous Glucose Monitoring; ATTD Advanced Technologies & Treatments for Diabetes; isCGM intermittent glucose monitoring; min: minutes; h: hours; mg: milligrams; dl: deciliter; HbA1c glycosylated hemoglobin; CV: Variation coefficient.

**Table 3:** Description of sex and age of the study patients categorized according to the type of insulin treatment.

	MDI (*n* 155)	CSII (*n* 36)	*p* Value
**Sex**			0.740
**Male**	50.9%	47.2%	
**Female**	49.9%	52.8%	
**Age**	11.218 (3.276)	10.782 (3.392)	0.323
**Mean (SD)**			

SD—standard deviation; MDI—Multiple doses of insulin; CSII—Insulin subcutaneous continuous infusion.

**Table 4:** Results of the variables obtained from CGM at 3 months and one year in patients with CSII or MDI.

	3 Months			1 Year		
	*CSII (n = 155)*	*MDI* (*n* = 36)	*p Value*	*CSII (n = 155)*	*MDI (n = 36)*	*p Value*
TIR 70–180 mg/dl	54.78% (12.74)	47.09% (15.46)	0.017	52.76% (11.49)	46.68% (15.39)	0.064
TIR 70–140 mg/dl	35.63% (9.68)	29.27% (11.85)	0.010	34.12% (11.30)	28.12% (11.40)	0.018
CV (%)	62.31% (78.37)	40.58% (7.84)	0.002	43.08% (4.42)	40.36% (6.41)	0.020
Number of scans	10.35 (4.73)	9.64 (5.99)	0.31	10.68 (4.63)	9.47 (4.94)	0.26
***TBR (<70 mg/dl)***	7.38% (3.44)	5.17% (4.56)	0.016	7.32% (4.38)	4.6% (4.00)	0.03

CSII: continuous subcutaneous insulin infusion. MDI: multiple doses of insulin. TIR: in range rate. CV: coefficient of variation. TBR: time below range. mg: milligrams; dl: deciliter.

**Table 5:** Patients categorized by the level of glycosylated hemoglobin at the beginning of the study and one year after the use of the sensor, divided according to the type of treatment used.

	Basal			1 Year		
	CSII (*n* 36)	MDI (*n* 155)	*p* Value	*CSII* *(n 36)*	*MDI* *(n 155)*	*p Value*
HbA1c ≤ 6.5%	36%	29%	0.543	35%	11.5%	0.013
HbA1c 6.5–7%	27.7%	25.8%		40%	23.1%	
HbA1c 7–7.5%	22.2%	19.3%		20%	35.9%	
HbA1c 7.5–8%	11.1%	15.4%		0%	16.7%	
HbA1c > 8%	3%	10.5%		5%	12.8%	

HbA1c glycosylated hemoglobin; CSII: continuous subcutaneous insulin infusion. MDI: multiple doses of insulin.

**Table 6:** Number of capillary blood glucose controls performed before the use of the continuous glucose monitoring system and one year after its use, categorized according to the different insulin treatment guidelines.

	MDI (*n* 155)	CSII (*n* 36)	*p* Value
Number of blood glucose levels before sensor use. Mean (SD)	6.774 (1.169)	8.324 (1.435)	<0.001
**Number of blood glucose levels one year after using the sensor. Mean (SD)**	1.070 (1.736)	1.044 (1.692)	0.249

CSII: continuous subcutaneous insulin infusion. MDI: multiple doses of insulin. SD: Standard deviation.

## Data Availability

All data are included in the tables of the article.
